# Clinical Practice of Endoscopic Submucosal Dissection for Early Colorectal Neoplasms by a Colonoscopist with Limited Gastric Experience

**DOI:** 10.1155/2013/262171

**Published:** 2013-12-11

**Authors:** Wen-Hsin Hsu, Meng-Shun Sun, Hoi-Wan Lo, Ching-Yang Tsai, Yu-Jou Tsai

**Affiliations:** Division of Gastroenterology, Department of Internal Medicine, Yuan's General Hospital, No. 162 Cheng-gong 1st Road, Lyngya District, Kaohsiung City 802, Taiwan

## Abstract

*Objectives*. Endoscopic submucosal dissection (ESD) for early colorectal neoplasms is regarded as a difficult technique and should commence after receiving the experiences of ESD in the stomach. The implementation of colorectal ESD in countries where early gastric cancer is uncommon might therefore be difficult. The aim is to delineate the feasibility and the learning curve of colorectal ESD performed by a colonoscopist with limited experience of gastric ESD. *Methods*. The first fifty cases of colorectal ESD, which were performed by a single colonoscopist between July 2010 and April 2013, were enrolled. *Results*. The mean of age was 64 (±9.204) years with mean size of neoplasm at 33 (±12.63) mm. The mean of procedure time was 70.5 (±48.9) min. The rates of *en bloc* resection, R0 resection, and curative resection were 86%, 86%, and 82%, respectively. Three patients had immediate perforation, but no patient developed delayed perforation or delayed bleeding. *Conclusion*. Our result disclosed that it is feasible for colorectal ESD to be performed by a colonoscopist with little experience of gastric ESD through satisfactory training and adequate case selection.

## 1. Introduction

Endoscopic submucosal dissection for early colorectal neoplasm has been gradually utilized and its safety and effectiveness have been shown in Japan, other Asian countries, and in the West [1–4]. Compared with endoscopic mucosal resection (EMR), ESD could be used to resect larger lesions in a whole piece and, therefore, affords more accurate pathological examination and less local recurrence rate [5, 6]. The development of ESD technique arose from the stomach and esophagus and finally to the colorectum and received consequent approval for the medical procedure from the Japanese insurance system. Compared with ESD in the stomach and esophagus, colorectal ESD is regarded as more risky and difficult. Therefore, it is recommended that at least 30 cases of gastric ESD should be completed before colorectal ESD is attempted [7, 8]. However, early gastric cancer cases are few in countries outside of Japan. Colorectal cancer remains one of the most common malignancies in the word. In Taiwan, it has been the cancer with highest incidence for the fifth consecutive year [9]. It is quite essential to use a less invasive technique to manage large adenomatous polyps or superficially invasive carcinoma of the colorectum. If the experience of gastric ESD is mandatory for colorectal ESD learning, it would be difficult for a colonoscopist outside of Japan to master this technique. We report our result of colorectal ESD performed by a colonoscopist with little experience of upper gastrointestinal tract ESD.

## 2. Method

### 2.1. Patients and Tumors

Colorectal ESD began to be performed at our endoscopy unit from July 2010. The inclusion criteria of colorectal ESD for our patients are (a) laterally spreading tumor, granular type, >2 cm; (b) laterally spreading tumor, nongranular type, >2 cm; (c) recurrent adenoma after previous polypectomy or EMR with size > 1 cm; (d) endoscopically diagnosed superficially submucosal invasion cancer with size > 1 cm; and (e) Subepithelial neoplasm, such as neuroendocrine tumor in the rectum with size between 1 and 2 cm. Informed consents were obtained before each ESD after clear explanation about the ESD process, risk, benefits, probable complications, and how to manage if any complications occur.

### 2.2. Colorectal ESD Procedure

All the colorectal ESD were performed by one colonoscopist (W-H. H.). Before the beginning of the colorectal ESD, the operator had completed 3,000 colonoscopies, 100 colorectal EMR, but merely one gastric ESD. The operator attended two sessions of* in vivo* and *ex vivo* porcine model for ESD training held by the Digestive Endoscopy Society of Taiwan. The operator also visited the National Cancer Center, Chuo Hospital in Tokyo, Kyoto Prefectural University of Medicine Hospital, and Osaka Medical Center for Cancer and Cardiovascular Diseases as a clinical observer for learning colorectal ESD technique and knowledge.

**Figure 1:**
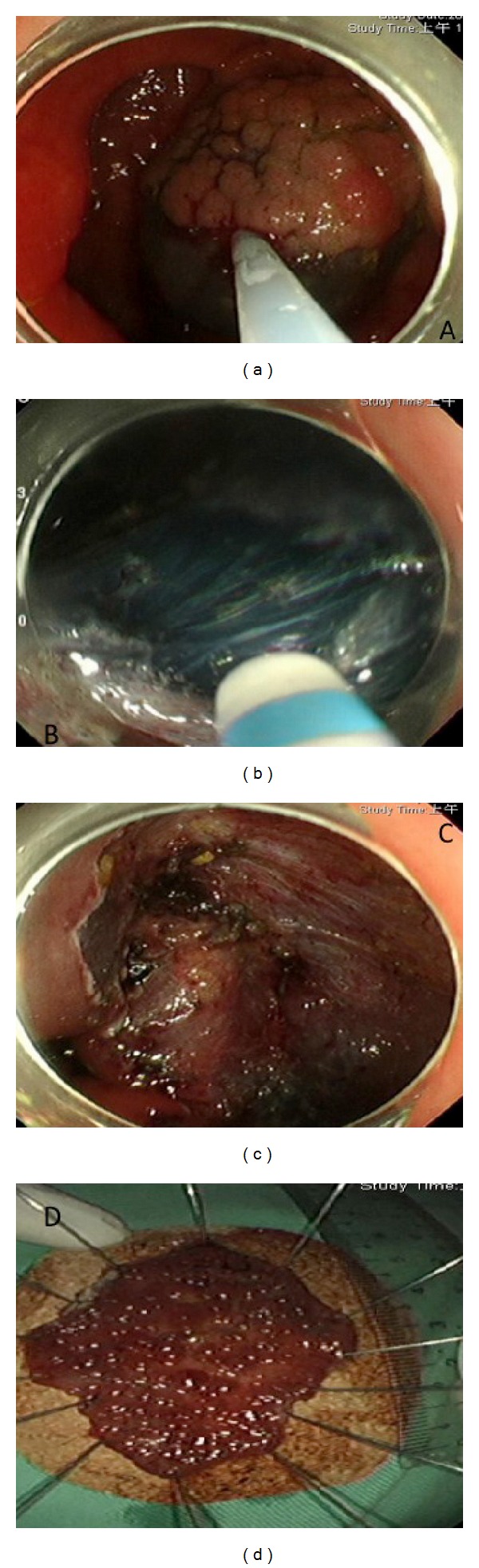
Process of endoscopic submucosal dissection. (a) A laterally spreading, granular, homogenous type tumor, 5.5 cm in diameter, was noted at ascending colon. Fluid was injected into submucosal layer to create cushion for mucosal incision and submucosal dissection. (b) Dual knife was used for submucosal dissection along the blue layer that was caused by indigo carmine being administered to enhance dissection plane. (c) The tumor was already resected *en bloc*. The artificially created ulcer base was checked carefully for muscular layer injury and exposed vessel. (d) The resected specimen was stretched and fixed well on a plate.

The patients were ordered onto a low-fiber diet the day before colorectal ESD. Bowel preparation was done with 2-liter polyethylene glycol plus 240 mg simethicone on the day of colorectal ESD. 20 mg butylscopolamine (Buscopan, Boehringer Ingelheim, Ingelheim, Germany) was given intravenously before and readministered during the procedure if necessary. Colorectal ESD were performed for all patients with clear consciousness, except one patient with a lesion at the junction of the descending-sigmoid colon who received 5 mg midazolam (Dormicum, Cenexi SAS, Fontenay-sous-Bois, France) intravenously during the ESD. A gastroscope with water-jet function was used for the first 10 cases including two lesions at proximal transverse colon (Olympus GIF-H260Z, Olympus, Tokyo, Japan) and a newly developed colonoscope was used for latter cases regardless of location (Olympus PCF-260AZI, Olympus, Tokyo, Japan). Soft straight distal attachment (D-201-12704, Olympus, Tokyo, Japan) was used to help maintain submucosal plain for dissection. The fluids for submucosal layer lifting and maintenance were glycerol and hyaluronate sodium solution mixed with 4 mL 0.4% indigo carmine and were injected by a 23-gauge needle (NM-200U-0423, Olympus, Tokyo, Japan). The electrosurgical knife used for mucosal incision and submucosal dissection was a dual knife (KD-650Q, Olympus, Tokyo, Japan) (Figure 1). In selected cases, an IT knife (KD-610L) was used to help submucosal dissection. Bleeding control was done with Coagrasper (FD-410LR or FD-411 QR, Olympus, Tokyo, Japan). The electrosurgical generator was ESG-100 with water pump AFU-100 (Olympus, Tokyo, Japan). The energy setting used for incision and dissection was “forced coagulation 2” 30–40 W, whereas “soft coagulation” 80–100 W was used for hemostasis. CO_2_ insufflation during the procedure was performed for all patients. The resected specimen was retracted by Spider-Net (ConMed, New York, USA). The procedure time was defined as the time between the beginning of the submucosal injection and the completion of the dissection. We also calculated the resection velocity, which was shown as the minutes spent in resecting per square centimeter (min/cm^2^).

### 2.3. Histopathology

The resected specimen was fixed on a plate and sent to the pathology department for fine slicing in 1 to 2 mm intervals. The pathology diagnosis was based on Vienna classification of gastrointestinal epithelial neoplasia [10]. The *en bloc *resection was that the lesion was resected as a whole piece. The R0 resection was defined when the resected specimen was revealed free of tumor in both vertical and lateral margins. The curative resection was achieved when the specimen revealed negative lateral and vertical resection margins and met three of all the following criteria: (a) If submucosal layer invasion was present, the depth was within 1,000 *μ*m; (b) No lymphovascular invasion; and (c) The cell type was not poorly-differentiated. Tumor budding within grade 0/1 was added to the criteria for curative resection after July 2012 at our pathology unit [11, 12].

### 2.4. Complication

The “immediate perforation” was defined as perforation occurring immediately during the procedure and the “delayed perforation” meant the perforation occurred after completion of the procedure. Delayed bleeding was defined as evident hematochezia or melena developed after completion of the procedure.

## 3. Statistics

Independent *t-*test was used to analyze the velocity difference between former and latter cases. Since the population size was small, the Mann-Whitney *U* test was also used. Statistics analysis was conducted by SPSS Statistics version 19.0 for windows. A *P* value < 0.05 was defined as the level of statistical significance.

## 4. Result

From July 2010 to April 2013, a total of 50 colorectal ESD were performed successfully in the enrollment period, excluding five patients who quitted and were referred for surgery due to severe fibrosis (Table 1). The mean age was 64 (±9.204, SD). The mean tumor size was 33.1 mm (±12.63, SD) (Table 1). The mean procedure time was 70.5 minutes (±48.9, SD). The rates of *en bloc* resection, R0 resection, and curative resection were 86%, 86%, and 82%, respectively. Three patients suffered immediate perforation (6%, 3/50; two at transverse colon, one at ascending colon) (Table 2). The first perforation case, developed at the very early phase of our colorectal ESD, underwent surgery and the latter two cases were treated successfully by clipping, nothing per os, and antibiotics. No delayed perforation or delayed bleeding was noted in our patients. Our mean resection velocity was 9.8 min/cm^2^ (±7.9). We divided our patients into former 20 cases/latter 30 cases or former 40 cases/latter 10 cases and compared the resection velocity of each group. The resection velocity at each period differentiation was not statistically significant, though the period of the first 20 cases had slower velocity (11.3 min/cm^2^, ±9.6). Subgroup analysis for each study period, tumor location, and macroscopic appearance was also performed (Table 3).

## 5. Discussion

The training and learning curve of colorectal ESD in Japan is clear [13, 14]. In addition to animal model training, at least 20–30 cases of gastric ESD are necessary before the colorectal ESD is attempted. Besides, it should begin from the rectum under the supervision of experts. Hotta et al. described a learning curve of 40 cases of colorectal ESD to prevent perforation and 80 cases to acquire skill for large colorectal tumors [15]. Sakamoto et al. also reported the learning curve from two trainees and showed that colorectal ESD could be performed safely and independently after completion of 30 cases [16]. However, operators in both studies were experienced with gastric ESD. The situation may change in countries with lower incidence of gastric cancer.

In the Western world, Probst et al. reported their feasible low complication rate at distal colon, although the resection rate was not as high as those from Japanese studies [3]. The learning curve showed that the *en bloc* resection rate, R0 resection rate, and procedure time improved during the study period. However, most of these cases were limited to the rectum and the learning curve may be unable to extrapolate to lesions at the right-sided colon.

In Asia, reports from Taiwan, Korea, and China about colorectal ESD also showed competent *en bloc* resection and complication rates [17–20]. Most of the colonoscopists, however, also had good experience in gastric ESD. The learning curve and whether the colorectal ESD could be performed by colonoscopists with little gastric ESD experience were not clear from these studies.

We analyzed our 50 cases with the division by several different intervals and found that the first 20 cases had slower resection velocity than the latter 30 cases. However, this was not statistically significant. Our results revealed that tumors located in the rectum had slower resection velocity. We assumed it would be due to the initial case selection effect. It is notable that there was no perforation case occurring in the rectum. Our experience found that it was easier for LST-G to maintain submucosal lifting and may therefore result in faster resection velocity in our study. It did not reach remarkable difference statistically, probably due to our small population. Nevertheless, our result confirmed the Japanese expert's recommendation for the case selection at initiation, that is, to start with laterally spreading, granular type tumors, 2-3 cm in diameter, located in the rectum [13, 21].

In view of the analysis for the *en bloc* resection and perforation rate, our result of colorectal ESD is not inferior to earlier studies [22–24], although it is seemingly worse than recent studies [25–27]. Hotta et al. also compared the resection velocity as the learning curve in that the resection velocity improved from 18.9 min/cm^2^ (1st period) to 12.6 min/cm^2^ (2nd period) [15]. Our mean resection velocity was 9.8 min/cm^2^ for the first 50 cases. We do not think our technique was more superior, but it represented an improvement of colorectal ESD technique after the efforts of Japanese experts. The development of new instruments, standardization of the technique, and hands-on training all contributed to the shorter procedure time, higher *en bloc* resection rate, and lower complication rate [28].

Sakamoto et al. mentioned unlooped colonoscopy insertion technique as a prerequisite to perform colorectal ESD [16]. That was to complete more than 10 cases of total colonoscopy within 5 min without causing abdominal discomfort in patients. Ohata et al. also mentioned skillful performance at total colonoscopies as one of the prerequisites before colorectal ESD [7]. Good colonoscopy insertion technique may represent skillful scope control that is a fundamental portion of ESD procedure. Compared with gastric ESD experience, masterful colonoscopy insertion technique may be more indispensable before colorectal ESD, although this may need to be proven more.

The limitation of our study is its single-operator and single-institution design. Our experience showed that good colonoscopy insertion technique may be important, but how to define a competent colonoscopist would be another issue. Furthermore, we think it is quite necessary to frequently inquire of Japanese experts about ESD problems and observe them demonstrating ESD. It is helpful to overcome a bottleneck during the learning of colorectal ESD.

## 6. Conclusion

Our result revealed that, through animal model training, acquaintance with the latest knowledge, learning from Japanese experts' live demonstration, and adequate selection of the initial cases, it is also feasible, effective and safe for colorectal ESD to be performed by a colonoscopist with limited experience of gastric ESD.

## Figures and Tables

**Table 1:** Patient and tumor characteristics.

	Total
Patients' characteristics, *n* (%)	
Total number of the cases	50
Sex, male/female	25/25
Mean age, years	64 (range: 46–82)
Lesions' characteristics	
Mean tumor size, mm	33.1 (range: 12–70)
Macroscopic type, *n* (%)	
LST-G	18 (36)
LST-NG	13 (26)
Protruded	19 (38)
Tumor location, *n* (%)	
Cecum	1 (2)
Ascending colon	6 (12)
Transverse colon	12 (24)
Descending colon	5 (10)
Sigmoid colon	10 (20)
Upper rectum (Ra)	8 (16)
Lower rectum (Rb)	8 (16)
Histopathology, *n* (%)	
Neuroendocrine tumor	3 (6)
Adenoma, low-grade dysplasia	28 (56)
Adenoma, high-grade dysplasia	6 (12)
Intramucosal carcinoma	7 (14)
Invasive carcinoma, SM1	1 (2)
Invasive carcinoma, SM2	3 (6)
Sessile serrated adenoma/polyp	2 (4)

LST-G: laterally spreading tumors, granular; LST-NG: laterally spreading tumors, nongranular; SM1: submucosal invasion <1,000 *µ*m; SM2: submucosal invasion ≥1,000 *µ*m; Ra: rectum above reflection line; Rb: rectum below reflection line.

**Table 2:** Results of endoscopic submucosal dissection for colorectum.

	Total
Procedure time, mean, min	70.5 (range: 16–240)
*En bloc* resection rate, *n* (%)	43 (86)
R0 resection rate, *n* (%)	43 (86)
Curative resection rate, *n* (%)	41 (82)
Immediate perforation rate, *n* (%)	3 (6)
Delayed perforation rate	0
Delayed bleeding rate	0

**Table 3:** Subgroup analysis for resection velocity.

Subgroup	Velocity, min/cm^2^	*P* value
Case 1~40/case 41~50	9.8/9.66	0.96
Case 1~20/case 21~50	11.26/8.79	0.32
Rectum/nonrectum	12.98/8.27	0.11
G/NG	7.73/10.95	0.39

G: granular; NG: nongranular.
